# Receptor Binding-Induced Conformational Changes in Herpes Simplex Virus Glycoprotein D Permit Interaction with the gH/gL Complex to Activate Fusion

**DOI:** 10.3390/v15040895

**Published:** 2023-03-30

**Authors:** Doina Atanasiu, Wan Ting Saw, Tina M. Cairns, Harvey M. Friedman, Roselyn J. Eisenberg, Gary H. Cohen

**Affiliations:** 1 Department of Basic and Translational Sciences, School of Dental Medicine, University of Pennsylvania, Philadelphia, PA 19104, USA; wsaw@upenn.edu (W.T.S.); tmcairns@upenn.edu (T.M.C.); ghc@upenn.edu (G.H.C.); 2 School of Medicine, University of Pennsylvania, Philadelphia, PA 19104, USA; hfriedma@pennmedicine.upenn.edu; 3 School of Veterinary Medicine, University of Pennsylvania, Philadelphia, PA 19104, USA

**Keywords:** HSV, glycoproteins, protein–protein interactions, conformational changes, antibodies

## Abstract

Herpes simplex virus (HSV) requires four essential virion glycoproteins—gD, gH, gL, and gB—for virus entry and cell fusion. To initiate fusion, the receptor binding protein gD interacts with one of two major cell receptors, HVEM or nectin-1. Once gD binds to a receptor, fusion is carried out by the gH/gL heterodimer and gB. A comparison of free and receptor-bound gD crystal structures revealed that receptor binding domains are located within residues in the N-terminus and core of gD. Problematically, the C-terminus lies across and occludes these binding sites. Consequentially, the C-terminus must relocate to allow for both receptor binding and the subsequent gD interaction with the regulatory complex gH/gL. We previously constructed a disulfide bonded (K190C/A277C) protein that locked the C-terminus to the gD core. Importantly, this mutant protein bound receptor but failed to trigger fusion, effectively separating receptor binding and gH/gL interaction. Here, we show that “unlocking” gD by reducing the disulfide bond restored not only gH/gL interaction but fusion activity as well, confirming the importance of C-terminal movement in triggering the fusion cascade. We characterize these changes, showing that the C-terminus region exposed by unlocking is: (1) a gH/gL binding site; (2) contains epitopes for a group (competition community) of monoclonal antibodies (Mabs) that block gH/gL binding to gD and cell–cell fusion. Here, we generated 14 mutations within the gD C-terminus to identify residues important for the interaction with gH/gL and the key conformational changes involved in fusion. As one example, we found that gD L268N was antigenically correct in that it bound most Mabs but was impaired in fusion, exhibited compromised binding of MC14 (a Mab that blocks both gD–gH/gL interaction and fusion), and failed to bind truncated gH/gL, all events that are associated with the inhibition of C-terminus movement. We conclude that, within the C-terminus, residue 268 is essential for gH/gL binding and induction of conformational changes and serves as a flexible inflection point in the critical movement of the gD C-terminus.

## 1. Introduction

The first essential step in HSV viral entry requires binding to a cellular receptor. The HSV receptor binding protein gD recognizes three distinct receptors: nectin-1, herpesvirus entry mediator (HVEM), and specific sulfate-modified forms of the glycan heparan sulfate [1]. Once gD binds to a receptor, fusion is carried out by three other viral glycoproteins: the heterodimer gH/gL (modulator of fusion) and gB (fusogen). Crystallography studies have shown that the C-terminus of the gD ectodomain (C-term) normally occludes the binding site for nectin-1 (gD is in a closed, autoregulated conformation) and prevents the formation of the N-terminal loop needed for HVEM binding [2,3,4]. For either HVEM or nectin-1 to interact with full-length gD (369 aa), the C-term residues of gD (roughly 265–316) must be displaced to expose the nectin-1 binding site, now considered to be an open conformation. As soluble proteins, gD_306t_ represents the closed conformation (non-binding) and gD_285t_ represents the open form (binding).

**Figure 1:**
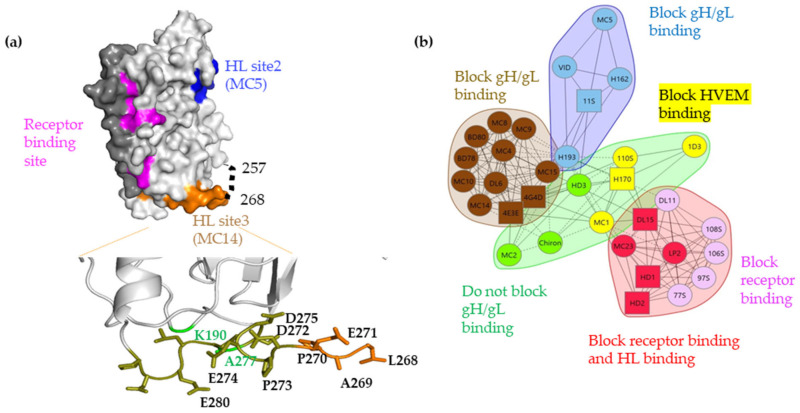
(**a**) Surface representation of gD306 crystal structure (PDB 2c36) showing the C-term (dark gray), the nectin-1 biding site (pink), and two of the three gH/gL binding sites defined by MC5 (site 2, blue) and MC14 (site 3, brown). gH/gL binding site 1 overlaps with the nectin-1 binding site. Region 257-267 which is missing from all crystal structures is shown as a dotted line and the C-term, lying over the receptor binding site. Point mutations generated in the gH/gL binding site 3 are shown as labelled side chains. In green are the point mutations that introduced the cysteine residues (at positions 190 and 277), which allow for the formation of a disulfide bond. (**b**) Based on competition and biological function, gD Mabs were grouped into communities: blue, brown, green, and red. The red community is subdivided into red and pink. The green community is subdivided into green and yellow. Circles indicate that competition was measured as both a ligand and an analyte; squares indicate that competition was measured as either a ligand or an analyte only. Solid connecting lines specify that competition between the two Mabs was measured in both directions (each as a ligand and analyte). Dashed connecting lines identify that the competition between Mabs was measured in one direction only.

The next step in the proposed fusion pathway is the interaction of receptor-activated gD with gH/gL protein complex. Recent evidence shows that gD interacts directly with gH/gL [5,6,7]. In that study, the physical interaction between gD and gH/gL was measured by surface plasmon resonance (SPR) when gD was presented as the shorter truncation (gD_285t_) in which the nectin-1 binding site is fully exposed [2,8]. We found that the gD–gH/gL interaction is blocked by specific neutralizing and virus spread blocking anti-gD monoclonal antibodies (Mabs), the epitopes of which define three putative gH/gL-interacting sites on gD: site 1 (MC23, mar 213), site 2 (MC5, 54, 75–77), and site 3 (MC14, 262–272) (Figure 1a). The location of site 3 is in a region termed “profusion” [9,10], hypothesized to be required for viral membrane fusion, and appears to function by recruiting other glycoproteins, namely gH/gL, to form a complex that triggers fusion [9,11]. The formation of the gD–gH/gL complex and triggering of the fusion cascade is highly dependent on specific conformational changes that occur in gD, beginning with receptor binding [12,13,14]. To further define these conformational changes, a gD mutant was engineered to contain an additional disulfide bond (K190C-A277C) which, based on the gD crystal structure, would constrain the motion of the C-term. This mutant (referred to as cys2) was able to bind both HVEM and nectin-1 [15], but failed to trigger cell–cell fusion and did not complement a gD-null virus, effectively separating the receptor binding and gH/gL interaction functions of gD. Together, these findings suggest that gD binding to a receptor is necessary but not sufficient for gH/gL binding and subsequent fusion. This mutant also helped define two physical “faces” on gD: a receptor binding face and a gH/gL binding face. Finally, the region in the gD cys2 protein that is locked by the newly introduced disulfide bond also hosts multiple epitopes targeted by protective antibodies (Figure 1b) [6,15] that block the physical interaction between gD and gH/gL [6], reduce viral spread [16] and protect against disease following passive immunization [16,17], further supporting a critical role for this region in transmission of the virus.

In this study, we address the underlying mechanism used by gD to transition from the “closed” form to the “open” conformation that (1) allow the transfer of information to gH/gL, (2) activate gH/gL, and (3) lead to induction of fusion. Specifically, we show that: (1) Binding of the nectin-1 receptor to the closed form of gD (gD_306t_) induces/allows the conformational changes required for gH/gL binding. (2) Unlocking of the disulfide bond in the gD mutant cys2 by reducing reagents enhances fusion due to unfettered movement of the C-term of gD, thus providing access for gH/gL to all three binding sites on gD. (3) Site directed mutagenesis of the gH/gL binding site 3 (262–282) on gD reveals epitopes of specific Mabs mapped to this region, and importantly, defines residues required for shifting from the closed to open state of gD. Together, our results delineate the conformational transitions induced in gD by the nectin-1 receptor for three key steps in fusion: (1) release of the autoregulation of gD; (2) full exposure of the nectin-1 binding site; and (3) rearrangements in gD structure that allow for the binding of gH/gL, all potential new targets for drug intervention.

## 2. Materials and Methods

**Cells.** Mouse melanoma cells B78H1 (gift from Meenhard Herlyn from the Wistar Institute, Philadelphia, PA, USA) were grown in DMEM containing 5% fetal bovine serum (FBS) and 100 µg/mL penicillin-streptomycin. For B78-C10 stably expressing nectin-1 [18], medium was supplemented with 500 µg/mL geneticin (G418). 293-T cells were grown in DMEM/10% FBS/100 µg/mL penicillin-streptomycin.

**Plasmids.** Wild-type glycoprotein constructs pTC580 (gB_2_), pTC578 (gD_2_), pTC510 (gH_2_), pTC579 (gL_2_), and Rluc8_(1–7)_, Rluc8_(8–11)_ for the fusion assay have all been described previously [19,20,21,22]. Full length gD2 DNA plasmids encoding point mutations N262A, Q265A, E267A, L268N, V269D, P270R, E271V, D272A, D275A, E280N, disulfide bonded cys2 mutant (K190C-A277C), and truncated versions of wt, L268N (285t and 306t), and Cys2 (306t) were generated by GenScript (Piscataway, NJ, USA) and cloned into pcDNA3.1 plasmid.

**Production and purification of soluble proteins.** Soluble nectin-1 [23] and gH_2_/gL_2_ [24] were purified from baculovirus-infected insect cells (Sf9) as previously described [25,26]. 6xHis tagged wt (285t, 306t), L268N (285t, 306t) and cys2 (306t) mutant proteins were purified from mammalian cells. 293T cells were plated in 6-well plates and transfected with 2 µg/well of each plasmid. Proteins were purified using Ni-nitriliacetic acid resin column and eluted with 250 mM imidazole.

**Antibodies used.** The following antibodies were used in this study: 1D3 [27], DL6, DL11 [28], MC2, 5, 10, 14, 23 [29], 4E3E (a gift from R. N. Laush).

**Split luciferase fusion assay.** The assay is described elsewhere [30,31]. Briefly, 5 × 10^4^ B78 cells (effector cells) were seeded on white, cell-culture treated 96-well plates. Then, 4 × 10^5^ C10 cells (target cells) were seeded on 6-well plates. Transfection was performed the following day. Briefly, a master mix containing 125 ng each of the gB, gH, gL, and Rluc8_(1–7)_ plasmids and 30 ng of either pCAGGS (mock), wt or mutant gD plasmid was split over three wells of effector cells. Target cells were transfected with 1 µg of Rluc8_(8–11)_ plasmid/per well. Twenty-four hours post-transfection, effector cells were pre-incubated for 1 h at 37 °C with EnduRen substrate (Promega, Madison, WI, USA) diluted 1:1000 in 40 µL fusion medium (DMEM without phenol red supplemented with 50 mM HEPES and 5% FBS). Target cells were detached with versene and pelleted. The pellet was resuspended in fusion medium and 40 µL of cells were transferred to effector cells, making the final volume 80 µL. Luciferase production was monitored over 2 h with measurements every 5 min using a BioTek plate reader.

*TCEP treatments*: Effector cells transfected as described were pre-incubated with 10 mM of freshly made TCEP for 10 min. Cells were washed with fusion medium and EnduRen substrate was added [31]. Fusion was triggered by the addition of target cells. A negative control (effector cell transfected with gB, gH, gL_,_ but no gD) was also included.

**Cell-based ELISA (CELISA).** B78 cells were seeded on clear 96 well plates (5 × 10^4^ cells per well). Transfection was done as described above for effector cells. Twenty-four hours post-transfection, cells were assayed for surface expression following a previously described protocol [31]. Briefly, 24 h post transfection cells were blocked with blocking solution (3% BSA in PBS^++^ containing Ca^2+^ and Mg^2+^) for 30 min. For detection, a 1 µg/mL of primary antibody and anti-rabbit or anti-mouse IgG HRP-linked secondary antibody (Cell Signaling Technology) were used. After addition of ABTS (2,2′-azinobis [3-ethylbenzothiazoline-6-sulfonic acid]) peroxidase substrate (Moss, Inc., Pasadena, MD, USA) absorbance at 405 nm was measured using a BioTek plate reader.

**Western blotting.** Here, 1 × 10^5^ C10 cells were seeded on 24-well plates and transfected with 125 ng of pCAGGS, gD wt or mutants. Forty-eight hours post-transfection, cells were washed 3 times with PBS and lysed (100 mM TRIS pH8, 1% NP40, 0.5% deoxycholate and 1× protease inhibitor). The protein concentration was measured using the Bradford assay. Twenty µg total lysate was run on Novex 10% tris-glycine gels, under “native” conditions [32]. After the transfer, nitrocellulose blots were probed with 1 µg/mL of the indicated antibodies. Secondary anti-rabbit/mouse IgG HRP-linked antibodies (Cell Signaling Technology, Danvers, MA, USA) were used, diluted to 1:2500. The blots were developed with ECL Western Blotting substrate or SuperSignal™ West Femto Maximum Sensitivity Substrate, both from Pierce (ThermoFisher Scientific, Waltham, MA, USA). Images were captured with Odyssey Imaging System (Li-Cor Biosciences, Lincoln, NE, USA).

Surface plasmon resonance (SPR). Experiments were performed using a Biacore 3000 biosensor (Cytiva, Marlborough, MA, USA), at room temperature. Filtered and degassed HBS-EP buffer (10 mM HEPES (pH 7.4), 150 mM NaCl, 3 mM EDTA, 0.005% surfactant P20) was used in all the experiments. An anti-His (Qiagen, Inc., Germantown, MD, USA) or anti-gD 1D3 antibody was covalently coupled to a CM5 sensor chip (Cytiva, Marlborough, MA, USA) following our previous protocol [33]. Then, 150–200 resonance units (RUs) of purified gD wt or mutants were captured by the antibodies. Purified IgGs and/or nectin-1 were then injected for 300 s, followed by gH/gL, also for 300 s. After each experiment, the chip surface was treated with brief pulses of 0.2 M Na_2_CO_3_ (pH 11) until the RU signal returned to baseline, and then a new cycle was started. All injections were performed at a flow rate of 5 μL/min.

## 3. Results

### 3.1. Binding of Nectin-1 Induces Conformational Changes in gD_306t_ (Closed Conformation) That Allow for gH/gL Binding

We previously showed that the open form of gD (gD_285t_) can physically interact efficiently with gH/gL [5,6] and could be blocked by specific gD and gH/gL Mabs. Notably, we had previously divided these Mabs into blue, red, and brown communities based upon their ability to compete for binding (Figure 1b), thus allowing us to refine the proposed protein–protein interaction sites on both gD and gH/gL [5,6]. We hypothesized that gD_306t_ would not physically interact with gH/gL in vitro because its conformation was locked in a closed position. However, if a conformational change in gD_306t_ is required to initiate the cascade, then the addition of soluble receptor nectin-1 in the SPR experiment should drive the C-terminus of gD away from the core, thus altering its conformation and allowing for gH/gL binding.

**Figure 2:**
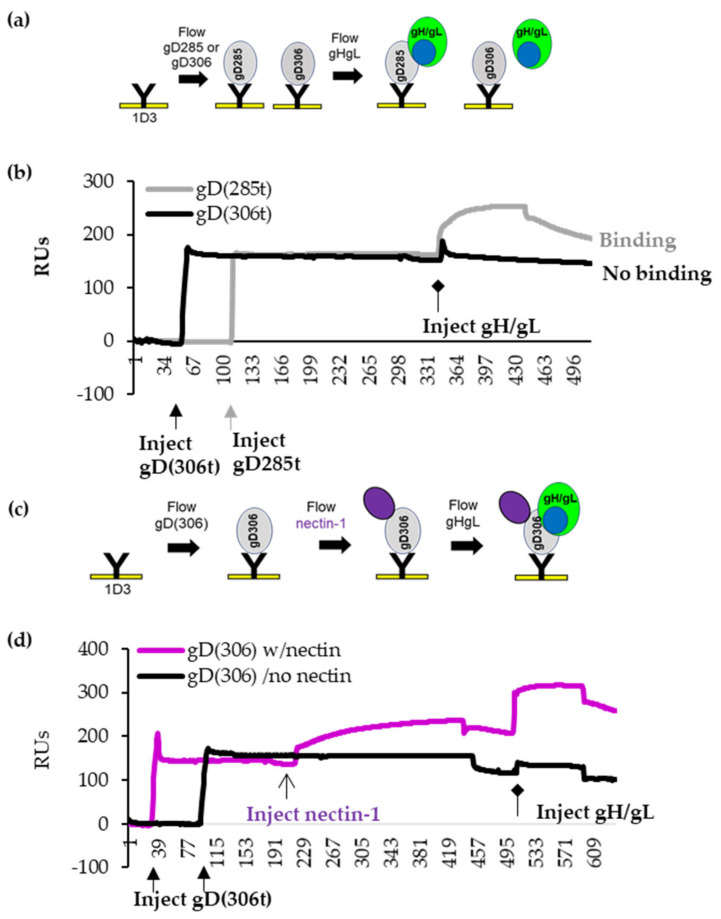
Binding of gHgL to gD by SPR. (**a**). Diagram outlining the SPR protocol. (**b**)**.** 1D3 (anti-gD Mab) was covalently coupled to a CM5 biosensor chip. An equal amount of gD_285t_ (gray) and gD_306t_ (black) were immobilized to different spots on the Biacore chip. Soluble gD_306t_ or gD_285t_ and gH/gL were sequentially injected (injection start indicated by diamond arrow). An increase in response units (RUs) after injection indicates binding. gH/gL binds to gD_285t_ only (black curve). No gH/gL binding was observed for gD_306t_ (gray curve). *x* axis, time (seconds). *y* axis, resonant units (RU). (**c**) Diagram of the SPR protocol for sequential injections of gD_306t_ and nectin-1, followed by injection of soluble gH/gL. (**d**) gD306 was attached to two flow cells on the biosensor chip via 1D3 Mab. Nectin-1 was flowed over cell 1 only. Flow cell 2 did not receive nectin. gH/gL was flowed over both flow cells. In the absence of nectin-1, gD_306t_ does not bind gH/gL (black curves). Pre-binding of nectin-1 to gD_306t_ induced conformational changes that allow for gH/gL binding (purple). Experiments were performed a minimum of three times.

To address this hypothetical model, we adapted the Biacore 3000 biosensor system (SPR) assay (Figure 2a). First, we amine-coupled anti-gD Mab 1D3 to a CM5 SPR chip. 1D3 Mab [34], which binds residues 11–21 in the N-terminus, captures both gD_306t_ (closed) and gD_285t_ (open), but importantly presents gD_285t_ in a conformation that allows for interaction with gH/gL interaction [5,6].

We used 1D3-coupled chips to capture equal resonance units (RUs) of gD_285t_ (open) and gD_306t_ (closed) (Figure 2b, thick arrows) and then flowed soluble gH/gL across the chip (Figure 2b, diamond arrowhead). gH/gL binding is indicated by the increase in RUs. As expected, gD_285t_ efficiently bound gH/gL (gray curve in Figure 2b), whereas gD_306t_ did not (black curve). Thus, the presence of residues 286–306 in the closed conformation of gD interferes with gH/gL binding.

Next, we captured gD_306t_ with 1D3 Mab and sequentially flowed nectin-1 to “open” gD and then gH/gL to assess the ability of gD to interact with gH/gL. Figure 2c shows the overview of a representative experiment. Nectin-1 binding to gD_306t_ is indicated by an increase in the number of RUs (Figure 2d, purple line). As shown in Figure 2b, the addition of gH/gL to gD_306t_ alone did not result in binding (no increase in mass) (Figure 2d, black line). However, gH/gL was able to bind to the nectin-gD_306t_ complex (Figure 2d, purple). This experiment shows that nectin-1 induced the necessary structural changes in gD that allow gH/gL to bind. Although displacement of the C-term of gD by receptor binding was expected to promote interaction with gH/gL based on the crystal structures of free and receptor-bound gD [2,8], this is the first time that these interactions were shown experimentally, albeit using soluble proteins.

### 3.2. Tethering of the C-Term by a Disulfide Bond in gD Prevents Movement Required for Fusion Function

We previously showed that cys2, a disulfide-bonded gD mutant (K190C-A277C; Figure 1a), could bind both nectin-1 and HVEM receptors but failed to trigger fusion [15]. This suggested that receptor binding was necessary but not sufficient to activate gD, and that additional changes in gD structure are needed for subsequent interaction with gH/gL (which at the time, could not be tested). As we are now capable of testing for gH/gL binding, we set out to determine whether the disulfide lock in cys2 restricted conformational changes in full length and/or soluble gD. We asked two questions: (1) what changes (if any) were induced by the presence of the disulfide bond in the putative gD–gH/gL interaction sites on gD and (2) how did the disulfide bond affect the fusion function of gD and its interaction with gH/gL?

**Figure 3:**
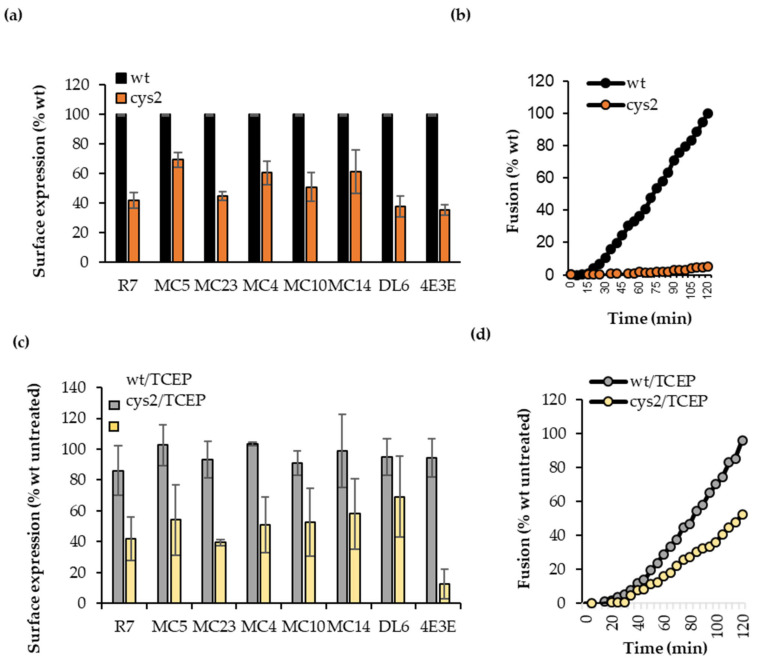
Characterization of wt and cys2 proteins. (**a**) CELISA of B78 cells transfected with full-length wt and cys2 gD and probed with select Mabs. Expression of cys2 (brown bars) is presented as % of binding to wt with the same antibody. The average of three experiments, each done in duplicate. (**b**) Fusion assay. Ability of full-length gD wt (black curve) and cys2 (brown curve) to trigger fusion in a live cell fusion assay. Data are presented as percent fusion by wt gD at the 2 h time point. Representative curve from three independent experiments, each done in duplicate. (**c**) CELISA after TCEP treatment. Binding of sentinel Mabs from each community to B78 cells transfected with full-length wt (gray) or cys2 (yellow) gD after cells were treated with TCEP for 10 min. Average of three experiments, each done in duplicate. Data for each antibody was normalized to no treatment wt samples. (**d**) Fusion assay. Effector cells expressing gB, gH/gL, and wt or cys2 gD were treated for 10 min with 10 mM TCEP before fusion was triggered by the addition of donor nectin-1 expressing cells. Data are presented as percent fusion by untreated wt gD at the 2-h time point, when the rate of fusion was at its maximum. Representative curve from three independent experiments, each done in duplicate.

To determine if gD conformation was altered by the presence of the disulfide bond, we used a cell-based ELISA (CELISA) to compare the antigenic structure of full length wt gD and cys2 mutant on the surface of B78 cells using Mabs MC5 (blue), MC23 (red) and MC4, MC10, MC14, DL6, and 4E3E (brown) (Figure 1b). The cys2 mutant is expressed at 40% of wt [15], as shown by a polyclonal gD antibody (R7), binding. As binding of our panel of Mabs was also at 40–60% of wt levels, the antigenic structure of the cys2 mutant appears similar to that of wt gD (Figure 3a) but is expressed at lower levels.

To test for the effect of the disulfide bond on fusion function, we used a split luciferase assay [31,35] in which effector B78 cells were transfected with the split luciferase RLuc_1–7_, gB, gH/gL, and gD, either as the wt or cys2 form. Donor C10 cells (constitutively expressing nectin-1) were transfected with the split luciferase RLuc_8-11_ plasmid. Upon co-cultivation of donor and effector cells, fusion is detected by reconstitution of the luciferase gene. As previously reported, we found that unlike wt gD (Figure 3b, black curve), cys2 was unable to trigger fusion (Ref. [15] and Figure 3b, brown curve).

### 3.3. Reduction of the Disulfide Bond with TCEP Restores Fusion Function of Cys2

We next set out to determine if relieving the “lock” on the movement of gD C-term restored gD fusion function. Reduction of artificially generated disulfide bonds successfully identified the residues essential for fusion triggering in measles H protein [36,37]. The bulky reducing reagent Tris(2-carboxyethyl) phosphine hydrochloride (TCEP) preferentially reduces the exposed disulfide bonds [38]. Wt gD carries three disulfide bonds that are essential for stabilization of its secondary and tertiary structure: Cys^66^-Cys^189^, Cys^106^-Cys^202^, and Cys^118^-Cys^127^ [2,39,40]. Notably, the native gD disulfides appear buried in the crystal structure [2,8,41], while the engineered Cys^190^-Cys^277^ bond appears completely exposed. Thus, preferential reduction of the engineered Cys^190^-Cys^277^ bond by TCEP would potentially “open” cys2 by freeing the C-term and allowing gH/gL to bind.

Before testing for gain of function, we used CELISA to determine whether TCEP treatment affects the general folding of the proteins. For this, B78 cells were transfected with full-length wt gD or cys2. Twenty-four hours post-transfection, cells were treated with 10 mM TCEP solution for 10 min. After treatment, cells were washed with PBS and CELISA was performed, as described. CELISA readings were normalized to the untreated wt samples probed with the same antibodies. TCEP treatment did not significantly change the antigenic structure of wt gD (Figure 3c, gray bars) or cys2 (yellow bars) compared to the untreated samples (Figure 3a). One exception was a loss of 4E3E epitope, which is consistent with conformational changes associated with this region of gD [6,34].

To test our hypothesis that the disulfide bond trapped gD in an intermediate conformation, we next determined if reduction of the artificial disulfide bond by TCEP led to a gain of fusion. For this, we used the cell-based fusion assay in which effector B78 cells were transfected with the split luciferase RLuc_1–7_, gB, gH/gL, and full-length gD, either wt or cys2. Donor C10 cells containing nectin-1 receptor were transfected with the split luciferase RLuc_8–11_. Twenty-four hours post-transfection effector cells were treated with 10 mM TCEP for 10 min at 37 °C. After treatment, cells were washed with fusion medium and incubated with EnduRen luciferase substrate for 1 h/37 °C. The fusion assay was carried out as described. The activity of both wt and cys2 was expressed as % of untreated wt gD. Figure 3d shows that the addition of TCEP did not affect the function of wt gD (gray curves). In contrast, TCEP treatment resulted in a gain in fusion function for full-length cys2 from 0 (Figure 3b) to 50% of wt activity, indicating that the cysteine substitutions themselves did not compromise the fusion activity of gD. We thus concluded that (1) the lack of function in cys2 was due to the disulfide lock introduced by the two new cysteine residues and (2) the reduction of the disulfide bond by TCEP treatment relieved the block, which resulted in a functional molecule.

### 3.4. Locking of C-Term to the Core of gD Allows for Nectin-1 Binding but Prevents the gD–gH/gL Interaction

To determine whether the inability of cys2 to function in fusion was specifically related to the inability of the locked C-term, associated with a lack of gH/gL interaction, we next determined whether the cys2 protein was capable of physically interacting with gH/gL by SPR. For this, soluble wt and cys2 gD proteins, truncated at position 306 and with 6xHis tags at the C-term, were purified by nickel column chromatography from supernatants of 293T transfected cells. Both wt and cys2 proteins were recognized by R7 anti-gD polyclonal antibody as well as by several sentinel Mabs representing different communities (Figure S1), confirming the correct folding of both proteins. Furthermore, as in our previous report using total cell lysates of cells transfected with full length constructs [15], cys2 soluble protein migrated faster on SDS-PAGE than wt (presumably due to the more compact structure adopted due to the disulfide bond).

**Figure 4:**
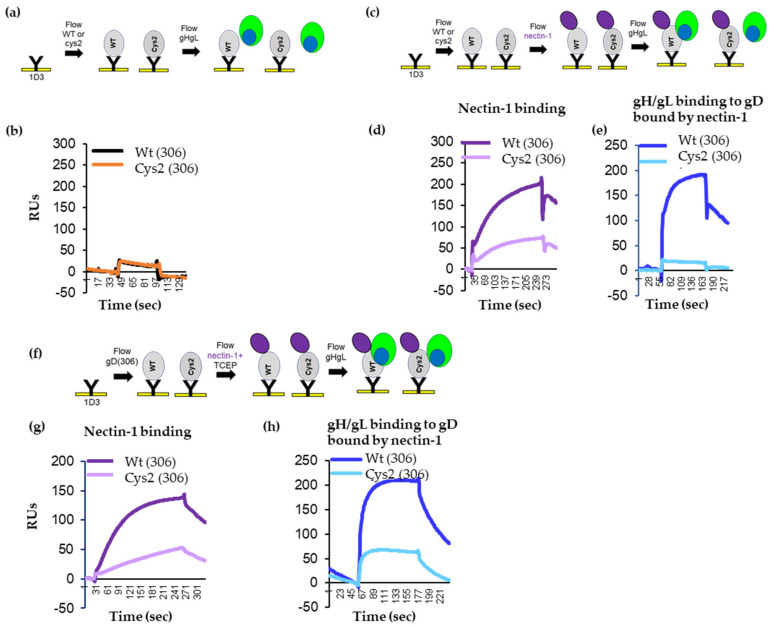
Characterization of wt and cys2 proteins. (**a**) Diagram of the Biacore 3000 protocol for analyzing gH/gL binding to gD, wt or cys2. (**b**) Soluble wt_306t_ and cys2_306t_ proteins were attached to the biosensor chip via 1D3 Mab. Soluble gH/gL was injected across the chip surface. Only the gH/gL binding curves are shown. Black curve, binding of gH/gL to wt gD; brown curve, binding of gH/gL to cys2. (**c**) Diagram of the Biacore 3000 protocol for analyzing the binding of gH/gL to a gD-nectin-1 receptor complex. gD molecules were attached to the biosensor chip via 1D3 Mab. Sequential injection of nectin-1 followed by injection of soluble gH/gL. (**d**) Nectin-1 binds to both wt (dark purple) and cys2 (light purple) gD proteins. (**e**) gH/gL bound to wt gD (blue) but not to cys2 mutant gD (cyan). (**f**) Diagram of the Biacore 3000 protocol for analyzing gD–gH/gL binding in the presence of TCEP. gD molecules were attached to the biosensor chip via 1D3 Mab. Nectin-1 was pre-mixed with 1 mM TCEP and flowed over the captured gD molecules, followed by the injection of soluble gH/gL. (**g**) Nectin-1 binds to both wt (purple) and cys2 (light purple) gD proteins. (**h**) As expected, gH/gL bound to wt gD (blue). Due to the reduction of the disulfide bond, gH/gL is now able to bind cys2 mutant gD as well (cyan).

To test for gH/gL binding, the truncated proteins were captured at their N-terminus on the surface of a CM5 chip via immobilized 1D3 antibody, as done in Figure 2. After capture, soluble gH/gL was flowed across the chip surface (Figure 4a). As expected, both gD_306t_ and cys2 bound gH/gL poorly (Figure 4b). We then asked whether nectin-1 would induce the necessary conformational changes in gD cys2 that allowed for gH/gL binding in wt gD. Both proteins bound nectin-1 (Figure 4d and [15]), albeit with different efficiencies (cys2 bound nectin-1 ~40% of wt). As shown in Figure 2d, after gH/gL was flowed, there was a significant binding of gH/gL to wt gD_306_ (Figure 4e, blue curve). However, there was no increase of gH/gL binding to cys2 (cyan). We thus concluded that, despite similarities to wt gD (antigenic profile, binding to receptor), the failure of cys2 to induce fusion may be linked to its inability to interact with gH/gL and that the disulfide bond prevented conformational changes in cys2 required for this interaction.

### 3.5. TCEP Treatment of Soluble Cys2 Truncated Protein Allows for the Interaction with gH/gL

To demonstrate that the gain-of-fusion function by cys2 after TCEP treatment (Figure 3d) was associated with a restored gD–gH/gL interaction, we repeated the SPR experiments in the presence of TCEP. Similar amounts of wt and cy2 gD proteins were immobilized on the 1D3-coupled chip. We then flowed soluble nectin-1 pre-mixed with 1 mM TCEP, followed by an injection of gH/gL (Figure 4f). The addition of TCEP did not affect the binding of nectin-1 to wt or mutant gD (compare Figure 3d,g). Furthermore, the nectin-1/TCEP mix did not change the ability of wt gD to bind soluble gH/gL (compare the blue lines in Figure 3e,h). In contrast, after nectin-1/TCEP treatment, gH/gL bound to cys2 (compare the cyan curves in Figure 3e,h). We propose that the soluble cys2 mutant failed to interact with gH/gL due to a locking of gD C-term and that the removal of the lock resulted in the opening of gD to a functional fusion competent molecule that could physically bind gH/gL and trigger fusion.

### 3.6. Site-Directed Mutagenesis in the 262–282 Region of gD Identifies Residues Important for gH/gL Binding

The engineered disulfide bond (Cys190-Cys277) links the core of gD to the postulated interaction site (262–282) with gH/gL (gH/gL site 3). This region contains multiple, critical epitopes that are recognized by Mabs from the brown community (Refs. [29,34,42] and Figure 1b) that block the physical gD–gH/gL interaction [6], inhibit fusion [7], block virus spread [16], and passively protect mice against herpes disease [16,43,44]. We hypothesized that these Mabs functions either work directly by blocking the physical gD–gH/gL interaction or indirectly by interfering with the movement of the C-term. We focused first on movement, as cys2 failed to bind gH/gL (Figure 4e) despite an intact MC14 epitope (gH/gL binding site 3) (Figure 3a and Figure S1). This suggested that MC14 might block the movement of the C-term and, in the process, affect the ability of gD to interact with gH/gL. To characterize the effect of point mutations on the movement involved in the opening of gD and its relationship to gH/gL binding, we generated 10-point mutations within the 262–282 region by site-directed mutagenesis. The expectation was that: (1) some mutants might show a defect in fusion activity; or (2) some mutations might change or inhibit the gD–gH/gL interaction. This would identify residues important for both the gD function and gH/gL interaction.

**Figure 5:**
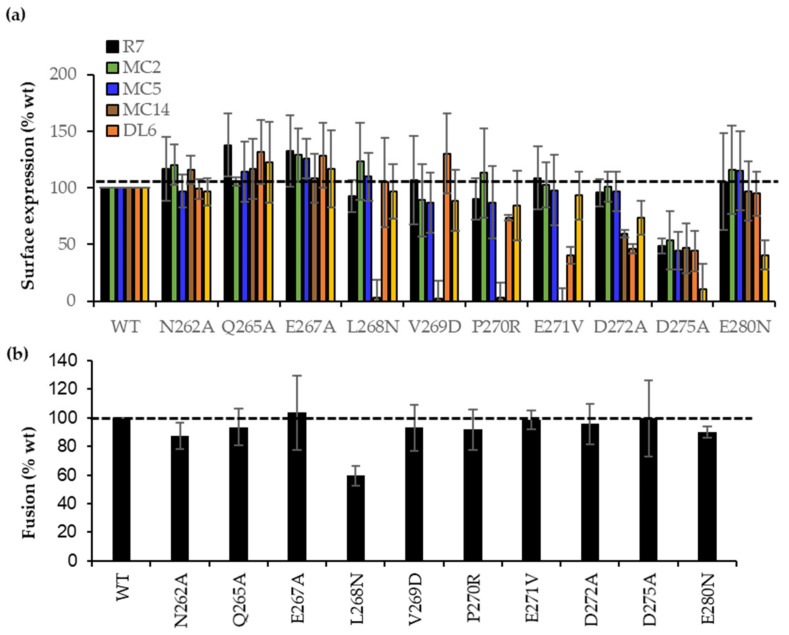
Characterization of point mutations in region 262–280 of full-length gD. (**a**) CELISA. Detection of the surface-expressed wt and point gD mutations with sentinel Mabs from each community. Data normalized to wt with each Mab (dotted line). Average of three experiments, each done in duplicate. (**b**) Fusion function of full-length wt and mutant gDs when combined with gB, gH, and gL constructs. Data normalized to wt gD (dotted line). Average of three independent experiments, each in duplicate.

CELISA data (Figure 5a) shows that the B78 cells surface expression level for all 10 mutant gDs was equivalent as measured by gD polyclonal antibody (Pab) R7 (black bars). Mabs MC2 (green bars) or MC5 (blue bars) recognized all constructs at wt levels. Thus, proper synthesis, processing, and transport of each gD mutant to the cell surface was unaffected. Notably, three Mabs from the brown community, MC14, DL6, and 4E3E (Figure 1b), which recognized overlapping peptides [34,42], differentially recognized gD (data summarized in Table 1). Briefly, L268N, V269D, P270R, and E271V mutants failed to bind MC14, but were bound normally by DL6 and 4E3E. The E271V, D272A mutants showed lower DL6 binding, and the D275A and E280D mutants failed to bind 4E3E, effectively mapping important residues in MC14, DL6, and 4E3E epitopes, respectively.

Next, we asked if the mutants impacted the functional interaction with gH/gL. Given the importance of this region in the gD–gH/gL interaction [6], we hypothesized that mutants deficient in the binding of brown antibodies would have a disrupted the gD–gH/gL interface, which would block the ability of gD to trigger fusion. For this, B78 cells were transfected with RLuc_1–7_, full-length wt, or mutant gDs alongside gB, gH, and gL. Figure 5b shows that most mutants had wt activity. Because V269D, P270R, and E271V had wt fusion phenotypes but failed to bind MC14, they fulfilled the concept of antibody-escape mutants [45]. Similarly, E271V, D272A, and D275A were escape mutations for DL6 and D275A, and E280N for 4E3E Mab. However, the L268N mutant showed 60% activity when combined with gB and gH/gL. The functional impairment of L268N, combined with its failure to bind MC14 (Figure 5a), suggests that the fusion impairment might be due directly to the gH/gL interaction rather than binding to the nectin-1 receptor.

### 3.7. Characterization of Soluble gD L268N

To distinguish between defects in receptor binding and altered physical interactions between gD and gH/gL, both wt and L268N gD soluble proteins were expressed and purified from the supernatants of transfected 293t cells. Each construct was expressed in two forms: the open 285t and the closed 306t. If the L268N mutation affected the conformational changes necessary to expose the nectin-1 binding site, then the open 285t protein should readily bind nectin-1 while the closed 306t protein should not. However, if the L268N protein was unable to interact with gH/gL, then neither the 285t truncation nor the 306t form should bind gH/gL, even after opening of the molecule with nectin-1.

By western blotting analysis, we found quantitatively similar binding of wt and mutant protein with the anti-His Mab. Notably, the L268N mutant protein consistently runs faster than expected (Figure S2a), suggesting that this mutation induces gD to adopt a more compact shape, similar to that of cys2 protein (Figure S1a). Second, we used SPR to determine the possible epitope alterations of the L268N mutant compared to that of wt gD. For this, wt gD and L268N mutant proteins were each presented via an anti-His Mab covalently bound to the chip. Non-competing sentinel antibodies from each community were then flowed sequentially over the chip. Wt and L268N gD proteins were tested in both the shorter (285t) and longer (306t) configurations. Similar to our findings with full-length proteins by CELISA (Figure 5a), we saw that except for MC14, soluble wt and mutant gD proteins bound each Mab with similar kinetics (Figure S2c). This confirms that the mutant gD protein is antigenically correct and that the L268N mutation introduces local changes that affect the MC14 binding only.

### 3.8. gD L268N Mutant Does Not Bind gH/gL

**Figure 6:**
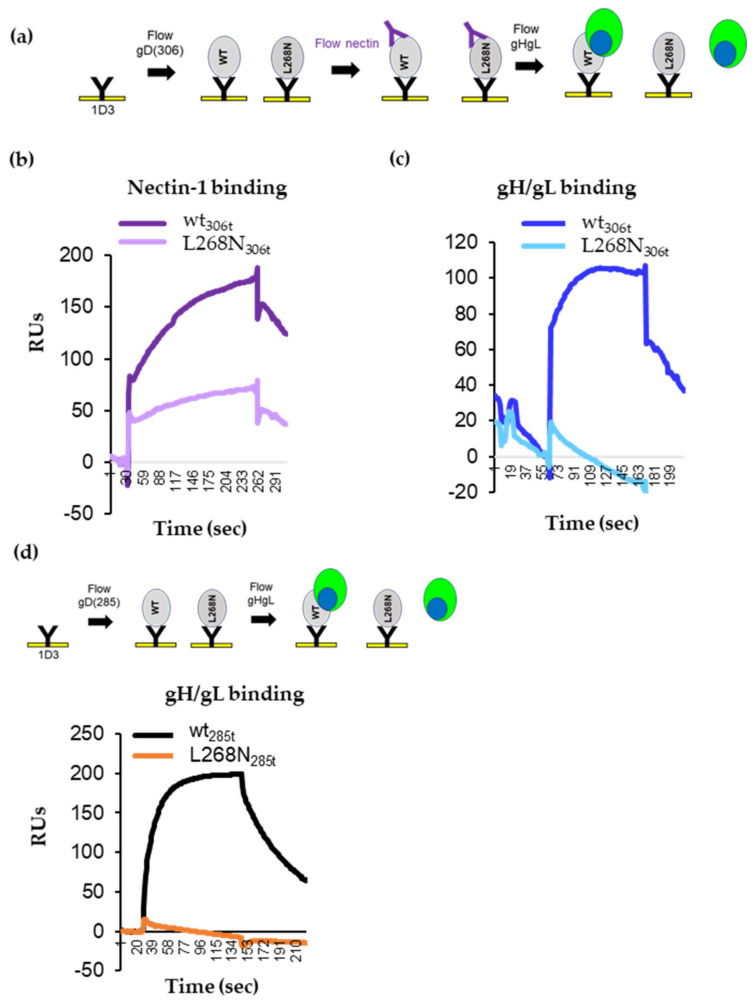
Ability of gD L268N to bind gH/gL. (**a**) Diagram of the Biacore 3000 protocol for analyzing the binding of gH/gL to a gD-receptor complex. gD molecules, wt, or L268N, both as 306t truncations were attached to the biosensor chip via 1D3 Mab. Sequential injection of nectin-1 followed by injection of soluble gH/gL. (**b**) Nectin-1 binds to both wt (purple) and L268N (pink) gD proteins. (**c**) gH/gL was flowed over the gD-nectin-1 complexes. gH/gL bound to wt gD (blue) but not to L268N mutant gD (cyan). (**d**) Diagram of the SPR protocol for analyzing gH/gL binding to gD_285t_, wt, or the L268N mutant. Only the gH/gL binding curves are shown. gH/L binds to wt gD (black curve). No gH/gL binding was observed for the L268N mutant (orange curve).

To determine whether the L268N mutation directly affected conformational changes necessary for nectin-1 binding or instead affected the subsequent gD–gH/gL interaction, we used SPR. First, similar amounts of the wt and mutant gD proteins were immobilized on the chip surface. Then, soluble nectin-1 was added, followed by soluble gH/gL (Figure 6a). Both wt gD_306t_ and gDL268N_306t_ bound nectin-1 (Figure 6b) albeit with different efficiencies (L268N bound nectin-1 ~50% of wt). After soluble gH/gL was flowed over the chip, only wt gD showed an increase in RUs (indicative of gH/gL binding) (Figure 6c, blue curve); mutant L268N_306t_ did not bind gH/gL (cyan).

Second, we looked at the binding of gH/gL to the open 285t truncation forms. Wt and L268N gD were captured by the 1D3 Mab anti-gD Mab that was covalently coupled to the surface of the chip. Then, soluble gH/gL was flowed over the chip. As expected from our previous studies (Figure 2b and [5,6]), wt gD_285t_ bound efficiently to gH/gL (the black curve in Figure 6d). In contrast, gD L268N_285t_ failed to bind gH/gL (orange curve).

Together, these results suggested that although wt and mutant gD were antigenically similar and could move the C-term to expose residues important for nectin-1 receptor binding, the conformational changes in L268N were not sufficient to allow binding to gH/gL.

**Figure 7:**
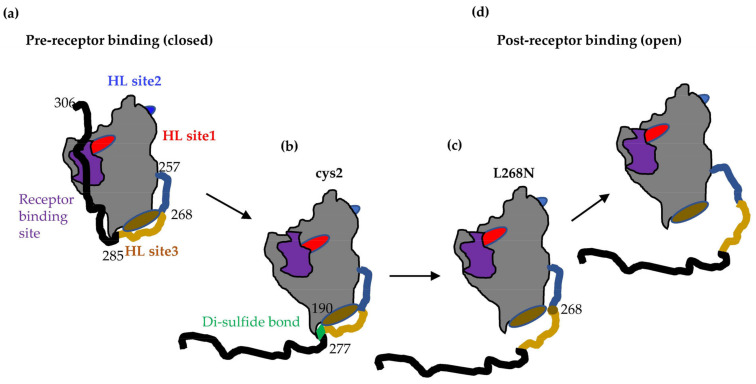
Proposed intermediates for the transition of gD from a pre-receptor (closed) to post-receptor binding state (open). (**a**) Before binding to a receptor, gD is in a closed conformation, with the receptor binding site (purple) covered by the C-term (black). The three gH/gL binding sites are also shown in red (site 1), blue (site 2), and brown (site 3). (**b**) In the presence of receptor or antibodies that compete with the receptor for binding, the C-term is displaced to expose the receptor binding site. Residue 277 is one inflection point in the C-term that is sufficient for receptor binding, but not gH/gL interaction. (**c**) Access to gH/gL binding site 3 (brown) is controlled by a second inflection point at position 268. Mutagenesis of this residue results in a molecule that partially exposes the gH/gL site 3. (**d**) After opening of gD past residue 268, gD is in a fully open conformation that can bind gH/gL.

We propose that the L268N mutation is essential for gD–gH/gL interactions by either directly affecting the gD–gH/gL interface or indirectly restricting the movement of the gD C-term. We hypothesize that there are multiple inflection points within the C-term of gD and that residue 268 acts as a fulcrum point that prevents specific, local conformational changes that restrict access to the gH/gL binding site 3 by introducing a rigidity in the C-term of gD (intermediate 2 in Figure 7), much like the disulfide bond in the cys2 gD mutant.

## 4. Discussion

HSV viral entry begins with the binding of gD to one of its cellular receptors and ends with the fusion of the two membranes mediated by the fusogen gB. While conformational changes in HSV gD were thought to be required for the initiation and execution of virus entry, supporting evidence was based on crystal structures and many presumed steps based on similarities with other viruses. In this study, we provide essential insight into the conformational changes that the HSV viral-entry glycoprotein gD undergoes following initial binding to nectin-1 that promote physical interaction with the modulator of fusion, gH/gL to promote fusion.

In the absence of its receptors, gD adopts a closed conformation (Figure 7a) in which the C-terminal region (residues 285–306) is anchored by Trp294 and contacts the N-terminal region (23–27). In this conformation, the C-term of gD covers the nectin-1 binding site and occupies the same space as the gD N-terminal residues in the gD-HVEM complex. As a result, the formation of either nectin-1-gD [41] or HVEM-gD complexes [2] requires similar conformational changes [8], with displacement of residues from the gD C-terminal region. This conformational change was hypothesized to serve as a signal to trigger the fusion cascade, as binding of gD to receptor would not only bring the viral and cellular membranes in close proximity but would also expose regions of gD that permit recruitment of gH/gL and/or gB, thus permitting fusion. The flexible, proline-rich C-terminal region of gD (260–285) was termed “the profusion domain” [9] and contains linear epitopes recognized by Mabs from the brown community (Figure 1a) that block viral spread and cell–cell fusion [7,16] and define one of the three gH/gL binding sites on gD [6].

The physical interaction between gD and gH/gL only occurs with the open form of gD (gD_285t_) ([5,6] and Figure 2b). As a longer gD truncation (gD_306t_/closed conformation), does not bind gH/gL, it suggests that the presence of a longer C-term interferes with gH/gL binding (Figure 2b). However, in the presence of nectin-1, the C-term of gD is displaced, which then allows for gH/gL to bind (Figure 2d). Thus, while the movement of gD C-term is necessary for receptor binding [2,8], it may also be required for physical interaction with gH/gL.

Notably, we find that a partial opening of gD that allows for nectin-1 binding is not sufficient to accommodate binding to gH/gL. Indeed, gD molecules that carry mutations at Trp294 or a linker insertion (∇290–299) are impaired in promoting virus entry and complement virus entry at levels of about 10% of wt, despite a more flexible C-term and an increased affinity for receptors [8,33,46,47]. The requirement for a full opening of gD is further exemplified by the gD cys2 mutant presented here. When the C-term is locked to the core of gD by the introduction of a disulfide bond (Figure 7b), gD does not complement the virus, does not trigger cell–cell fusion (Ref. [15] and Figure 3b), and does not bind gH/gL (Figure 4e), despite the ability of nectin-1 to displace the C-term and access the binding site (Ref. [15] and Figure 4d). Removal of the lock in the presence of reducing agents restores gH/gL binding and converts gD to a fully functional molecule (Figure 3d, and Figure 4h). This demonstrates that the opening of the C-term needs to be wide enough to minimally expose the region locked by the disulfide (past residue 277) and that the movement exposes a region in gD essential for the gH/gL interaction.

A portion of gD upstream of the cys2 disulfide bond is missing from all the crystal structures available, suggesting a high degree of flexibility in this region. It was hypothesized that this region (256–268) could potentially serve as a hinge that moves in response to receptor binding [15]. Mutagenesis in the 262–275 region identified residue L268 as playing an important role in the function of gD. The full-length version of the mutant gD was only impaired in fusion. However, the truncated versions resembled the cys2 mutant in that they were unable to physically bind gH/gL or trigger cell–cell fusion (Figure 5b and Figure 6c) despite displacement of C-term by nectin-1 binding (Figure 6b). However, while the open conformation of wt gD_285t_ allows it to bind gH/gL even in the absence of a receptor [5,6], gD-L268N_(285t)_ was surprisingly unable to bind gH/gL (Figure 6d). This suggests that substitution of Leu268, which is located in the gH/gL binding site 3, is either: (1) essential for the gD–gH/gL interaction; or (2) induces conformational changes necessary for the gH/gL binding. Preliminary data suggest that the main limitation observed with gD-L268N is a decreased mobility of the C-term that results in an occlusion of gH/gL binding site 3 (Figure 7c).

We have also shown that movement of C-term of gD_306t_ can occur not only in the presence of nectin-1 [2,8], but can also be driven by specific Mabs. A consequence of this movement is to increase the affinity of gD_306t_ for receptors to levels similar to those measured for the open gD_285t_ [6,29,34], and to increase binding of gH/gL to gD_306t_. (Figure 7d). Preliminary data suggest that MC2 can move the C-term of gDL268N_306t_ sufficiently to increase binding of MC14 Mab (located in gH/gL binding site 3) but does not allow for gH/gL to bind.

We propose that the high degree of flexibility of region 257–267 is related to gD function and that Leu268 serves as in inflexion point in this region. Changes at this position reduce the flexibility of gD C-term and prevent conformational changes that allow for gH/gL binding. This suggests that although gH/gL binding site 3 was defined by Mabs that block this interaction (brown community, Figure 1b), these Mabs do not block the gD–gH/gL interaction directly, but rather prevent further conformational changes that would allow access to gH/gL site 3. While Mabs from the brown community do not neutralize the virus, they do block the virus spread of the cell–cell fusion and provide significant protection against herpes disease in animal models [48], highlighting the potential physiologic requirement of gD movement in HSV disease.

We conclude that the unravelling of C-term from the core of gD, beginning with Trp294 and extending all the way to Ala277, is sufficient for receptor binding [8,15]. However, further opening of gD beyond Leu268 is necessary to trigger fusion and allow full access to the gH/gL binding site 3. This study not only brings experimental proof for the conformational changes that occur in gD and are necessary for function, but also offers new targets for vaccine design and therapies.

## Figures and Tables

**Table 1:** Summary of the point mutations generated in region 262–280.

Mutation	Ab Recognition (%wt)					Fusion Activity (%wt)
	MC2	MC5	MC14	DL6	4E3E	
N262A	120 ± 17	97 ± 14	115 ± 14	100 ± 8	96 ± 12	80 ± 22
Q265A	105 ± 4	114 ± 26	116 ± 26	130 ± 28	122 ± 35	105 ± 12
E267A	128 ± 23	125 ± 17	108 ± 21	128 ± 28	116 ± 33	88 ± 8
L268N	123 ± 34	109 ± 21	3 ± 15	104 ± 39	96 ± 23	59 ± 9
V269D	88 ± 31	87 ± 26	2 ± 15	130 ± 35	88 ± 27	120 ± 29
P270R	87 ± 35	72 ± 30	2 ± 13	84 ± 2	88 ± 30	100 ± 18
E271V	102 ± 20	97 ± 31	1 ± 12	40 ± 7	93 ± 21	98 ± 8
D272A	100 ± 13	96 ± 17	60 ± 3	46 ± 4	73 ± 15	87 ± 19
D275A	53 ± 25	44 ± 16	46 ± 21	44 ± 17	10 ± 22	81 ± 18
E280N	115 ± 40	115 ± 45	96 ± 20	94 ± 3	40 ± 10	85 ± 10

## Data Availability

Not applicable.

## Supplementary Materials

The following supporting information can be downloaded at: https://www.mdpi.com/article/10.3390/v15040895/s1, Figure S1: Characterization of cys2 soluble protein. Figure S2: Characterization of soluble gD L268N.
