# Supplementary material for: Receptor Binding-Induced Conformational Changes in Herpes Simplex Virus Glycoprotein D Permit Interaction with the gH/gL Complex to Activate Fusion
Source: Viruses. 2023 Mar 30;15(4):895. doi: 10.3390/v15040895 (PMC10144430; doi:10.3390/v15040895)
Supplement: Supplementary file 1 [file viruses-15-00895-s001.zip › viruses-2306192-supplementary.pdf]

(a)

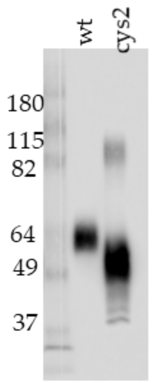

(b)

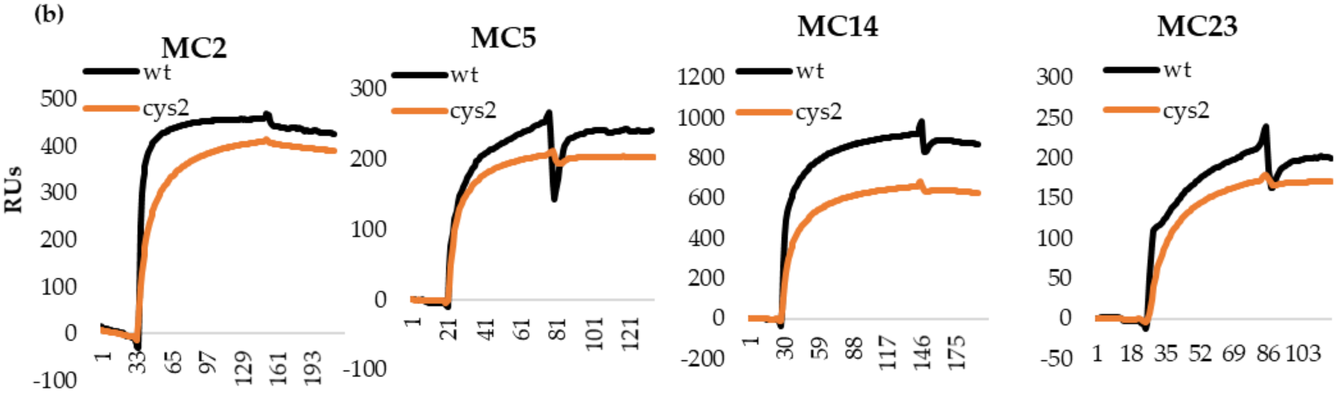

Figure S1. Characterization of cys2 soluble protein. A. Western blotting: R7 an anti- gD polyclonal Ab recognizes both wt and cys2 soluble proteins. B. Binding of sentinel Mabs by SPR. Equal amount of proteins were captured on the chip surface by 1D3 and a series of non-competing gD Mabs were sequentially flowed. All Mabs recognize both wt (black) and cys2 (orange) similarly.

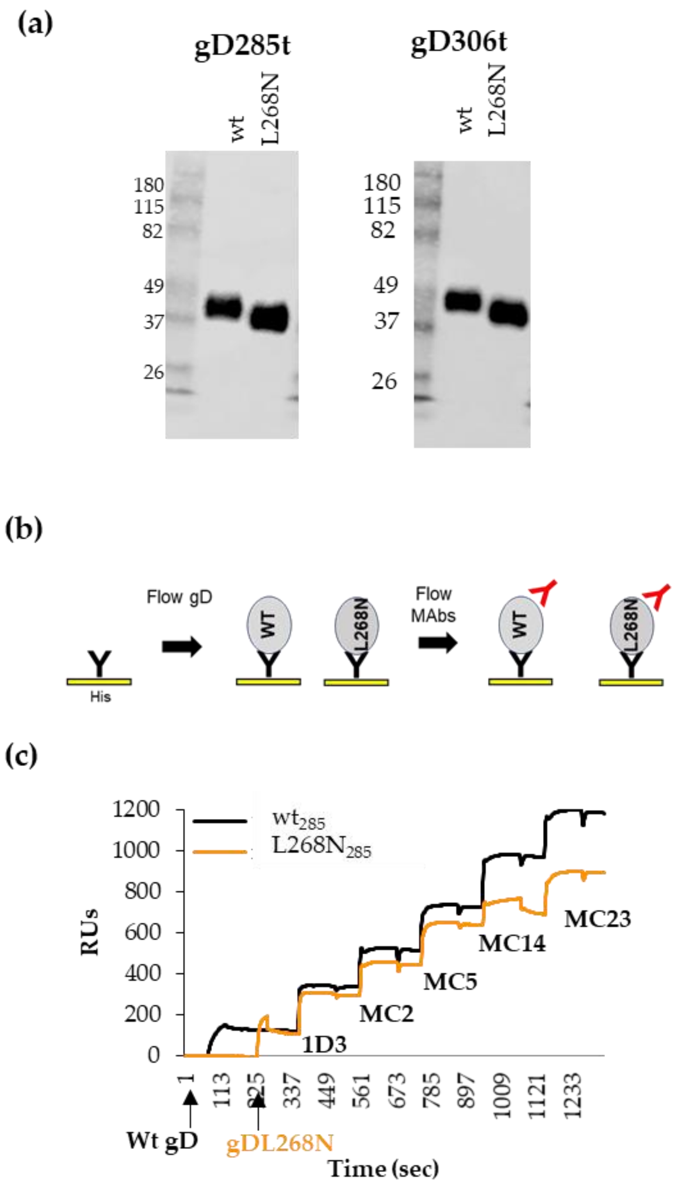

Figure S2. Characterization of soluble gD L268N. A. Western blotting of soluble wt and L268N mutant gD. Each protein was truncated and expressed as shorter (285t) or longer truncations (306t). Each His-tagged protein was run on a 10% SDS PAGE gel. Membranes were probed with anti-His Mab. Consistently, L268N runs faster than wt, regardless of the truncation point. B. Diagram of the Biacore 3000 protocol for determining the antigenic profile of wt and L268N gD mutant. C. Equal amounts of wt and L268N soluble proteins were attached to the biosensor chip via an anti-His Ab. Non-competing Mabs were sequentially injected across the chip surface. The chip was monitored for antigen-antibody binding, which would result in a step-wise increase in RUs. Binding curves: binding of Mabs to wt gD (black curves) or L268N (orange curves).
